# Selenium and the Methionine Sulfoxide Reductase System

**DOI:** 10.3390/molecules14072337

**Published:** 2009-07-01

**Authors:** Derek B. Oien, Jackob Moskovitz

**Affiliations:** Department of Pharmacology and Toxicology, School of Pharmacy, University of Kansas, Lawrence, KS 66045, USA

**Keywords:** selenoprotein, methionine sulfoxide, oxidative stress, antioxidants, posttranslational modification

## Abstract

Selenium is a chemical element participating in the synthesis of selenocysteine residues that play a pivotal role in the enzymatic activity efficiency of selenoproteines. The methionine sulfoxide reductase (Msr) system that reduces methionine sulfoxide (MetO) to methionine comprises the selenoprotein MsrB (MsrB1) and the non-selenoprotein MsrA, which reduce the *R*- and the *S-* forms of MetO, respectively. The effects of a selenium deficient (SD) diet, which was administrated to wild type (WT) and MsrA knockout mice (*MsrA^-^/^-^*), on the expression and function of Msr-related proteins are examined and discussed. Additionally, new data about the levels of selenium in brain, liver, and kidneys of WT and *MsrA^-^/^-^* mice are presented and discussed.

## Introduction

Accumulative posttranslational modification to proteins, mediated by the action of reactive oxygen species (ROS), is thought to be one of the major causes of aging and age-related diseases. Thus, mechanisms have evolved to prevent or reverse these protein modifications. While most protein damage by ROS is irreversible, methionine sulfoxide (MetO) in proteins can be reversed to methionine by the methionine sulfoxide reductase (Msr) system (consists of MsrA which reduces *S*-MetO and MsrB which reduces *R*-MetO, thioredoxin reductase (Trr), thioredoxin (Trx), and NADPH) [1,2]. The action of the Msr system may prevent irreversible protein damage, contribute to the cellular antioxidant resistance, and as a consequence extend organisms’ life spans. Evidence for the possible functions of the Msr system is demonstrated by the hypersensitivity to oxidative stress and shorter life span, as shown in several organisms lacking the MsrA protein [3,4]. A major biological role of the Msr system is suggested by the fact that the *MsrA* knockout mouse (*MsrA^-^/^-^*) is more sensitive to oxidative stress, accumulates higher levels of carbonylated protein and has a shorter life span than wild type mice [5]. Furthermore, overexpression of MsrA in human T cells, plant, and flies protects them from oxidative stress toxicity and leads to an almost doubling of the life span of flies [6,7,8]. In addition, selenium and MsrA are positive expression-regulators of the selenoprotein form of MsrB (MsrB1) and Trr [5,9]. Selenoprotein is defined as a protein that contains at least one selenocysteine residue or non-covalently bound selenium molecule (found in bacterial proteins). Examples of selenoproteins are: MsrB1, Trr, Gluthathione peroxidase (GPx), selenoprotein P (SelP), and selenoprotein W (SelW).

## Selenium deficient diet and *MsrA* knockout mice

Weaned pups of first mouse generation (F1) of wild type (WT) and *MsrA^-^/^-^* mice, fed with a selenium deficient (SD) diet, exhibited higher protein-MetO and carbonyl levels detected as early as six months of age [9]. Weanling mice were fed a SD torula yeast-based diet or a selenium-adequate (SA) diet containing 0.015 or 0.25 ppm selenium as Na_2_SeO_4_, respectively. Diets were prepared by Zeigler based on their SD torula yeast diet (Zeigler Brothers, Gardner, PA). Accordingly, it was concluded that selenium deficiency shortens the time required to cause significant accumulation of faulty proteins due to age-related oxidative stress. Previously, it was shown that exposing mice to 100% oxygen (hyperoxia) caused higher elevation of protein-carbonyl [5]. However, feeding mice with a SD diet probably better mimics physiological condition of oxidation, as a SD diet can lower the levels of active of selenoproteins, some of which have antioxidant functions (e.g. MsrB, Trr, and PGx). Thus, the SD diet enables the follow up on the appearance of oxidative posttranslational protein-modification (such as protein-MetO) especially in the *MsrA^-^/^-^* mice. 

After completion of the SD diet at the F1-generation, the mice are limited in their selenium consumption after they are weaned as they acquire selenium through their mother’s milk during the weaning period. However, continuation of the SD diet through the F2 generation will cause the selenium deficiency to start at birth (as the mother’s milk will be already deficient in selenium). Indeed, after completion of the SD diet at F1 generation, no significant observed phenotype has been noted in the *MsrA^-^/^- ^*relative to WT mouse, except for enhanced its atypical walking pattern (‘tip toe’ walking) [9]. Continuing the SD diet through the F2 generation caused slower body weight gain in *MsrA^-^/^- ^*mice till 120 days of the diet (that started immediately after weaning) [10]. This phenotype may imply that a significant decrease in the levels of certain selenoproteins (including MsrB and Trr) in conjunction with the absence of the MsrA protein may interfere with normal body growth at early stages of development. The observed phenotype may be related to the insufficient reduction of MetO residues in key proteins participating in early stages of mouse growth. Accordingly, it will be of great interest to identify the specific proteins in which MetO alters their ability to regulate directly or indirectly the function of growth related enzymes. 

The MsrA protein positively regulates Trr expression (especially under oxidative stress conditions) [5] and MsrB (under non-stress conditions) [9]. After the completion of SA diet, the most significant decrease in MsrB activity observed was mostly in liver and kidney tissues of *MsrA^-^/^- ^*mice [10]. However, only after completion of SD diet through the F2 generation, the *MsrA^-^/^- ^*mouse cerebellum showed a dramatic decline in MsrB activity in compare to WT brain cerebellum [10]. Consequently, it was suggested that a long-term SD diet exacerbates the negative affect on MsrB expression in the absence of MsrA only in cerebellum. It is possible that in cerebellum the expression levels of MsrA and selenium tightly regulate the expression level of MsrB to avoid excess MsrB synthesis when the potential for full reduction of protein-S-MetO is limited. Moreover, high levels of *S*-MetO and low levels of selenium may be secondary signals for reducing the synthesis of MsrB. 

It is apparent that enhanced protein oxidation occurs in the *MsrA^-^/^- ^*mouse tissues after the completion of a SD diet through the F2 generation, when compared to the F1 generation, and more so relative to WT mouse tissues [10]. The MsrA protein is highly abundant in brain cerebellum and alveolar macrophages in lungs [11]. Both cerebellum and alveolar macrophages require a high level of antioxidant defense to maintain their proper function. It is hypothesized that lower levels of selenium reduce the basal transcriptional level of selenoproteins with antioxidant properties (including MsrB1) and consequently lead to a compromised antioxidant defense resulting in enhanced protein oxidation. Based on these observations, it was concluded that a lack of MsrA exacerbates the effects caused by prolonged selenium deficiency.

The mammalian Trr enzyme is a selenoprotein that plays an important role in antioxidant defense and is also a major component of the Msr system. Moreover, MsrA is positively up regulating Trr expression under oxidative stress conditions [5]. As a result, both mouse strains (WT and *MsrA^-^/^- ^*mice) that were fed prolonged SD diet (through the F2 generation) demonstrated a dramatic decrease in their Trr activity, mainly in liver and kidneys [10]. The observed effects of the SD diet on Trr activity are again much stronger than the *MsrA^-^/^- ^*effects, except in brain where the opposite is true (when both *MsrA^-^/^- ^*mice and SD diet are applied through the F2 generation the effect of MsrA absence on Trr activity is stronger) [10]. One possible explanation is that very low selenium levels in brain contribute to the enhancement of oxidative stress that in turn is negatively affecting Trr expression especially in the absence of MsrA; similarly to the effect of hyperoxia on Trr levels in *MsrA^-^/^- ^*mice [5]. The direct cause for this Trr activity reduction in *MsrA^-^/^- ^*mice fed with a SD diet is yet to be determined. Thus, lowering Trr activity by both dietary-selenium and lack of MsrA, may play a significant role in fostering oxidative damage to proteins [10]. 

The reduction of Trx by Trr requires NADPH. The majority of NADPH is produced via the pentose-phosphate pathway, which is initiated by glucose-6-phosphate dehydrogenase (G6PD). Mouse embryonic stem cells and yeast cells lacking G6PD (by genetic manipulations) are more sensitive to cell death that is mediated by oxidative stress [12,13]. Hearts and lungs of *MsrA^-^/^- ^*mice that were fed the SD diet through the F2 generation showed significantly elevated expression and activity of their G6PD, relative to control WT mice [10]. Both lungs and heart are the first organs that are exposed to high levels of oxygen. Therefore, it is important to maintain sufficient reduction power (like NADPH) to prevent premature cell-death in response to extensive oxidative stress conditions. It is possible that the elevation of G6PD in these tissues (subjected to selenium deficiency) serves as a compensation mechanism for the lower antioxidant defense system in the *MsrA^-^/^- ^*mice, under conditions of prolonged SD diet. In is important to note that neither a SD diet nor a lack of MsrA alone showed this effect. The mechanism in which the up-regulation of G6PD occurs in the *MsrA^-^/^- ^*mice is yet to be discovered. It may be that the signal-mediators for this phenomenon are a combination of certain levels of cellular selenium and MetO (as free or protein-bound) that initiate signal transduction cascade leading to higher transcription level of *G6PD*. 

One selenoprotein that is considered to be a good marker for selenium deficiency is the secreted protein SelP [14]. Indeed, After the completion of a SD diet through the F2 generation, both mouse strains (WT and *MsrA^-^/^- ^*mice) exhibited very low levels of plasma SelP that were below the assay detection limits [10]. However, contrary to expectations, the cellular levels of SelP were significantly higher in the *MsrA^-^/^- ^*mice compared to control [10]. Usually, the SelP protein is secreted from cells to the plasma, and one of its possible roles is to deliver selenium to tissues via its rich selenocysteine residues content [14,15]. Another possible function of SelP is to act as an antioxidant by its potential reducing activity. SelP contains several redox centers in the form of cysteine and selenocysteine residues. Saito *et al*. have shown that the human SelP can catalyze the oxidation of reduced gluthathione (GSH) by a phosphatidylcholine hydroperoxide [16]. Additionally, Trx was found to be a very good substitute for GSH as a reducing agent [17]. If indeed the elevation of SelP level in the *MsrA^-^/^- ^*mice reflects a compensatory mechanism to accommodate higher cellular oxidative toxicity, it will suggest that SelP may have an important role as an antioxidant. Finally, support these observations regarding the level of expression of the glycosylated form of the cellular SelP, the non-glycosylated form of SelP in plasma, and its possible role in protecting against oxidative stress comes from the recent finding demonstrating SelP functions as an antioxidant [18]. Therefore, it will be interesting to crossbreed the *MsrA^-^/^- ^*mouse with *SelP^-^/^-^* mouse to create a double knockout *MsrA^-^/^- ^*/* SelP^-^/^-^* mouse and monitor for abrogated phenotypic characterization of either original source parent mouse (*MsrA^-^/^- ^*or *SelP^-^/^-^* mice). It is expected that oxidative stress related phenotypes will be enhanced in *MsrA^-^/^- ^SelP^-^/^-^* mice relative to each of the parent strain. 

In summary, the recent study on *MsrA^-^/^- ^*mice and a SD diet showed significant oxidative damage to proteins as a consequence of a long term SD diet through the F2 generation [10]. The enhanced accumulation of posttranslational modifications in the *MsrA^-^/^- ^*mice may be due to a compromised antioxidant defense. Not all tissues are equally affected by the selenium restriction. However, to compensate for the partial loss of selenoproteins that function also as antioxidants, certain tissues may up-regulate the activity and expression of specific proteins that are involved in reduction or peroxidation processes (e.g. G6PD and SelP). 

Unlike SelP and more like Trr, the activity of the selenoprotein and antioxidant enzyme GPx sharply declined in brains and livers of *MsrA^-^/^- ^*mice subjected to the SD diet through the F2 generation [10]. One possible explanation is that similarly to Trr, GPx expression is also under the control of MsrA especially under oxidative stress conditions mediated by prolonged selenium deficiency. The levels of GPx were not dramatically altered after the completion of F1 generation of the SD diet when compared with the F2 generation (up to ~ 50%, depending on the tissue), probably due to relative short time of the diet administrated right after weaning (40 days immediately after 21 days of weaning). The F2 generation of the SD diet enabled a dramatic effect of the SD diet and it is likely due to deficiency of selenium during the weaning period.

Among all tested tissues, only the cerebellum showed a major combined decrease in specific activities of MsrB, Trr, and GPx. This observation may reflect on a possible cerebellum malfunction associated with enhanced oxidative stress. It has been already noted that *MsrA^-^/^- ^*mice exhibited an atypical “tip-toe” walking pattern that is exacerbated as a consequence of a SD diet [5,9]. Since the cerebellum is also responsible for certain motor functions, it is possible that this form of ataxia is at least partially due to the significant loss of the above antioxidant activities. Also, it was shown that marginal zinc deficiency has a little effect on Msr system and that the oxidative effects of limited L-buthionine sulfoximine treatment did not up-regulate Msr activity [19].

Further investigations will be required to determine the identity of the oxidized proteins resulting from the SD diet. The gathered data will enable following signal transduction pathways and key proteins that are involved in the cellular regulation of oxidative stress that is associated with selenium metabolism. 

## Selenium content in organs of *MsrA^-^/^-^* in comparison with wild type control mice

In light of the increased sensitivity of the *MsrA^-^/^- ^*mice to effects of a SD diet, we thought it was important to quantify the basal levels of selenium in the three major tissues where MsrA is relatively highly expressed in normal mice. To address this issue, we have applied neutron activation analysis (NAA) to determine the content of selenium in both wild type and *MsrA^-^/^- ^*tissues (brain, liver, and kidneys). 

**Figure 1:**
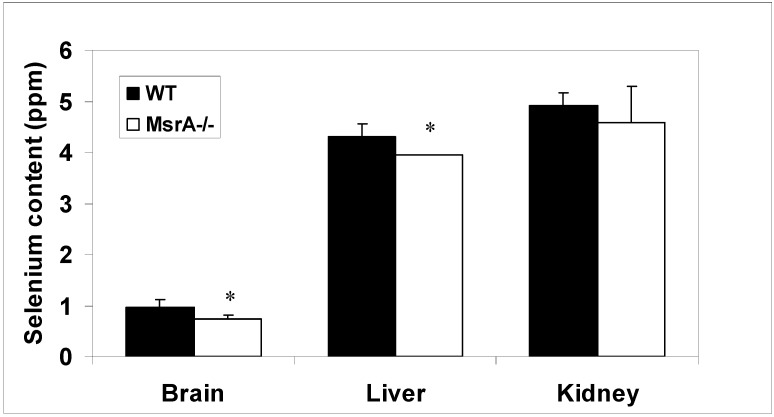
Selenium content in wild type and *MsrA^-^/^-^* mice. The selenium content (ppm) was measured by the relevant NAA method [20,21] in brain, liver and kidney using the same dry mass tissue from each organ. The quality control Sample was NIST SRM 1577 Bovine Liver (certified Se concentration = 1.1 +/- 0.1 ppm). The symbol * represents significant difference (*p*=0.05; *t-test*) between the averaged values of 3 independent experiments determining selenium content in tissues of wild type and *MsrA^-^/^- ^*mice (significantly lower selenium levels were observed in brain (-23%; *p*=0.05) and liver (-8%; *p*=0.043) of *MsrA^-^/^- ^*when compared with WT mice, respectively). Note: in the *MsrA^-^/^- ^*liver all measurements gave the same value; thus, no standard deviation is given.

The procedure to determine selenium in tissues is a based on the previously describe method by McKown and Morris [20], and that was modified according to Spatevl *et al*. [21]. The mouse tissues were air-dried and their masses were determined prior to the NAA analysis, performed by Dr. Morris at the University of Missouri-Columbia Research Reactor Center (Columbia, MO). The quality control sample was NIST SRM 1577 Bovine Liver (certified Se concentration = 1.1 +/- 0.1 ppm). As shown in Figure 1, the average selenium content in liver and kidneys is about 4-fold higher than in brain of both mouse strains (WT and *MsrA^-^/^- ^*mice). However, significantly lower selenium levels were observed in the brain (-23%; p=0.05) and liver (-8%; p=0.043) of *MsrA^-^/^- ^*when compared with WT mice, respectively. Accordingly, it is suggested that the MsrA-/- brain is more vulnerable to selenium deficiency mediated by SD diet [10] due to its relative lower basal selenium content. The fact that selenium is a trace element needed for selenoproteins’ function, any mild changes in its levels may affect the normal function of biological processes within an organ, and most importantly in brain. This does not imply that other more abundant elements are less important for normal cellular performance.

## Concluding Remarks

Currently, limited information is available regarding the importance of selenium to the Msr system components that require selenium to their activity, namely Trr and MsrB1. However, from the data of the current literature, it is apparent that feeding a SD diet may affect the function of these enzymes (Trr and MsrB1). Furthermore, lack of the *MsrA* gene exacerbates the accumulations of protein-methionine sulfoxides and protein-carbonyls when prolonged administration of a SD diet occurs. Accordingly, it is suggested that low selenium levels may reduce the antioxidant capability of the cell, especially when accompanied by low expression levels of antioxidant genes of the Msr system. Since the activities of the Msr system decline with age, while oxidative stress is increased, it could be beneficiary to increase the intake of selenium at an older age as a compensatory measure for the loss of antioxidants. It is clear that further research is needed to gather more knowledge on how selenium availability is affecting the structure/function of selenoproteins that are associated with the Msr system in brain. 
